# Surviving medicine and motherhood

**DOI:** 10.51866/mol.566

**Published:** 2024-01-11

**Authors:** Ak Lis Esther Sumi

**Affiliations:** 1 MB BCH BAO, MAFP, FRACGP, Klinik Kesihatan Tanah Puteh, Jalan Kwong Lee Bank, Kuching, Sarawak, Malaysia. Email: er8598@gmail.com

**Keywords:** Motherhood, Medicine, Family

First and foremost, the author would like to express her appreciation to the following three formidable FMSs who played a significant role in shaping her journey:

Dr Lenny Martini Hamden, who witnessed her evolution from the early days as an aimless medical officer; Dr Maila Mustapha, her trusty mentor during the treacherous endeavour of pursuing the daunting fellowship examinations; and Dr Peter Jerampang, her sporting gazettor, whose zest is her daily dose of caffeine.

My journey to joining the prestigious family medicine community was anything but easy. Juggling between motherhood and an ambition to pass my postgraduate examinations, posed formidable challenges. Each role was demanding but the unique challenges and joys they brought have defined my journey. Amidst chaos in trying to keep a delicate balance between medicine and motherhood, I unearthed some nuggets of wisdom:

1. **Caring for myself**
  Self-care became my holy grail. Scheduling time for myself, I found recreational activities to be vital for maintaining sanity. Fitness activities and gardening became my refuge, along with the occasional doses of retail shopping.
2. **Prioritising and planning ahead**
  The power of saying ‘no’ dawned upon me: ‘No’ to undertaking unnecessary responsibilities that do not align with my priorities. Mastering the art of role-switching became my superpower — from a dedicated family physician to a devoted mother in the flick of a switch. I embraced the mantra of planning ahead but being flexible enough to dodge life’s curveballs.
3. **Delegating**
  Delegation is not a sign of weakness but a stroke of genius. At home, letting my supportive husband share the household load and hiring a food caterer for cooking duties became my secret weapon to maintain sanity. At work, sharing responsibilities among colleagues tactfully fostered an atmosphere of teamwork and camaraderie.
4. **Setting realistic expectations**
  Perfection is mere illusion, and life’s priorities vary. I learnt to set my own pace and measure success in my own time and space. The societal and cultural pressure to be perfect all the time in every sphere of life: as a professional, parent and person can be daunting. However, not striving for the same things in life does not make one’s personal pursuits less valid.
5. **Counting my blessings**
  The privilege of caring for patients from ‘womb to tomb’ is a noble responsibility I hold dear. I do not always have it all together and am still trying to figure out the enigma called ‘balance’. However, I believe it is not impossible to have a satisfying career in medicine while staying engaged in the lives of my little ones.

Therefore, I keep my spirits high. For in this beautiful chaos of life lies the essence of my extraordinary journey.

**Figure:**
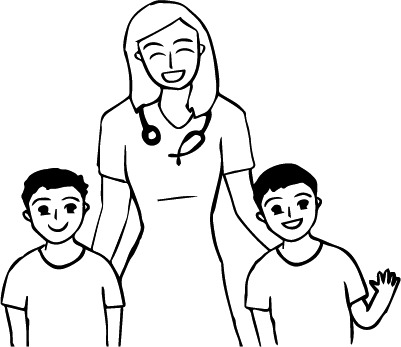
Portrait of the author and her boys. Illustration by Dr Samuel Kana Lis from Ren Ci Hospital, Singapore.

**Disclaimer:** The author is a young family medicine specialist (FMS), nearing the grand finale of her gazettement. She is also a proud and occasionally frazzled mother of boisterous preschool aged twins.

